# The impact of transportation infrastructure on bicycling injuries and crashes: a review of the literature

**DOI:** 10.1186/1476-069X-8-47

**Published:** 2009-10-21

**Authors:** Conor CO Reynolds, M Anne Harris, Kay Teschke, Peter A Cripton, Meghan Winters

**Affiliations:** 1 Institute for Resources, Environment and Sustainability, University of British Columbia (UBC), 2202 Main Mall, Vancouver, BC, V6T 1Z4, Canada; 2 School of Population and Public Health, UBC, James Mather Building, 5804 Fairview Avenue, Vancouver, BC V6T, 1Z3, Canada; 3 School of Environmental Health, 2206 East Mall, UBC, Vancouver, BC, V6T 1Z3, Canada; 4 Department of Mechanical Engineering, UBC, 2054-6250 Applied Science Lane, Vancouver, BC, V6T 1Z4, Canada

## Abstract

**Background:**

Bicycling has the potential to improve fitness, diminish obesity, and reduce noise, air pollution, and greenhouse gases associated with travel. However, bicyclists incur a higher risk of injuries requiring hospitalization than motor vehicle occupants. Therefore, understanding ways of making bicycling safer and increasing rates of bicycling are important to improving population health. There is a growing body of research examining transportation infrastructure and the risk of injury to bicyclists.

**Methods:**

We reviewed studies of the impact of transportation infrastructure on bicyclist safety. The results were tabulated within two categories of infrastructure, namely that at intersections (e.g. roundabouts, traffic lights) or between intersections on "straightaways" (e.g. bike lanes or paths). To assess safety, studies examining the following outcomes were included: injuries; injury severity; and crashes (collisions and/or falls).

**Results:**

The literature to date on transportation infrastructure and cyclist safety is limited by the incomplete range of facilities studied and difficulties in controlling for exposure to risk. However, evidence from the 23 papers reviewed (eight that examined intersections and 15 that examined straightaways) suggests that infrastructure influences injury and crash risk. Intersection studies focused mainly on roundabouts. They found that multi-lane roundabouts can significantly increase risk to bicyclists unless a separated cycle track is included in the design. Studies of straightaways grouped facilities into few categories, such that facilities with potentially different risks may have been classified within a single category. Results to date suggest that sidewalks and multi-use trails pose the highest risk, major roads are more hazardous than minor roads, and the presence of bicycle facilities (e.g. on-road bike routes, on-road marked bike lanes, and off-road bike paths) was associated with the lowest risk.

**Conclusion:**

Evidence is beginning to accumulate that purpose-built bicycle-specific facilities reduce crashes and injuries among cyclists, providing the basis for initial transportation engineering guidelines for cyclist safety. Street lighting, paved surfaces, and low-angled grades are additional factors that appear to improve cyclist safety. Future research examining a greater variety of infrastructure would allow development of more detailed guidelines.

## Background

Bicycling is an active mode of transportation that integrates physical activity into daily life. The bicycle is an attractive alternative to the automobile as an urban mode of transport. Cycling is associated with a range of individual and public health benefits such as improved physical and mental health, decreased obesity, and reduced risk of cardiovascular and other diseases [1-6], as well as ancillary benefits such as reduced emissions of noise, air pollutants and greenhouse gases [7,8]. There are significant economic costs of physical inactivity [9], and benefit-cost analyses suggest that the benefits of increased cycling are worth approximately four to five times the costs of investing in new cycling infrastructure [10,11]. These potential benefits suggest that it is important to increase the use of the bicycle as a mode of active transportation.

It is clear that the health benefits of cycling are significant, and at this point there is no reason to assume that health risks outweigh those benefits. However, a full public health understanding requires that attention be paid not only to long-term population health and environmental benefits of bicycling, but also to the factors that influence risk of injury and fatality. Bicyclists are vulnerable because they must frequently share the same infrastructure with motorized vehicles, and yet bicycles offer their users no physical protection in the event of a crash. In addition, the mass of a typical automobile is at least an order of magnitude greater than a bicycle plus its rider, and motorized vehicles have top speeds that are considerably faster than bicycles. As a result, bicycle riders who are involved in a crash are exposed to a much higher risk of injury compared to motor vehicle users (with the exception of motorcycle riders).

To date, most studies of cycling safety - especially in North America - have emphasized helmet design, regulation, and implementation to mitigate the severity of cycling injuries when a crash occurs [12,13]. This is particularly true for children [14,15]. In many North American jurisdictions children who cycle (and sometimes also adult cyclists) are required by law to use helmets, although this is not the case in most European countries. While helmets are effective in reducing the severity of head injuries, they do not address impacts to other parts of the body [16,17]. More importantly, they do not prevent incidents from occurring in the first place [18], and legislating their use may even discourage cycling [19].

The built environment has been implicated as an important determinant of bicycling rate [20-23], but these relationships are complex and a positive correlation has not always been found [24]. It is equally important to understand how the built environment affects bicycling safety because there may be an opportunity to prevent injuries by modifying transportation infrastructure. Infrastructure improvement meets several important conditions for successful injury prevention measures: (a) it is population based, rather than requiring initiative on the part of the individual; (b) it is passive, rather than requiring active participation; and (c) it is accomplished with a single action, rather than requiring repeated reinforcement [18].

In this paper we review the evidence on how different types of transportation infrastructure affect bicyclists' safety. This paper is organized as follows: first we provide an overview of bicycling safety and ridership. Next we offer definitions of, and alternative terminology for, the transportation infrastructure used by cyclists that might be expected to influence their safety (Table 1). We describe our literature search methodology and the inclusion and exclusion criteria, and present the results of the search in two detailed tables. Table 2 describes studies that assess the safety of intersections for cyclists, and Table 3 describes studies related to straightaways (i.e. roads, lanes, paths). We conclude by discussing the findings of this review, critiquing the methodological approaches used, and offering recommendations for future research.

**Table 1:** Key terminology for describing transportation infrastructure used by cyclists

Term	Description
STRAIGHTAWAYS	
On-road cycling/vehicular cycling	When bicyclists ride on a roadway designed primarily for motor vehicles.

Wide curb lane	The outer (curbside) lane of a paved multi-lane road is wider than the standard width and can accommodate cyclists, although there may not be signs indicating this.

Sharrows * (suggested cycle lane†)	Symbols painted on the paved roadway indicating that bicycles can share the lane with motor vehicles. They are sometimes used on roads with high cyclist traffic that don't have enough width to accommodate a bike lane.

Bike route	A paved residential or local road that is signed as being a "bike route", and may have cyclist-friendly crossings at major roads, such as traffic signals with push-buttons that are easily operated by cyclists.

Bike lane (marked cycle lane†)	Part of the paved roadway marked with painted lines or a colored surface, to designate that it is reserved exclusively for cyclists. Bike lanes may terminate before an intersection, or continue through it.

Cycle track (separated cycle lane†)	Paved lane, exclusively for bicycle use, next to a major city street or roundabout, but separated by a curb or other physical barrier.

Bike path	Off-road paved or unpaved path or trail, for bicycles only.

Multi-use path	Off-road paved or unpaved path or trail, shared with other non-motorized users (e.g. pedestrians, runners, or in-line skaters).

Sidewalk	Off-road paved walkway for pedestrian use, located by the side of road; known as "pavement" in some parts of the world (e.g. UK and Ireland).

Speed bumps/humps *	Raised ridge across the road designed to slow motor vehicle traffic ("traffic calming"), particularly in residential areas. Speed humps are easier than speed bumps for cyclists to ride over because they are less steep-sided and more broad.

INTERSECTIONS	
Intersections	Where two or more roads either meet or cross at the same level.

Junctions *	May be road intersections, but the term is usually used to refer to the point where a laneway, path, or driveway meets a road.

Roundabout	Intersection of arterial streets with a central circle of sufficient diameter that the road curvature accommodates all road vehicles, including trucks and buses. Roundabouts usually have splitter islands on the approaches, sidewalks around the edges, and crosswalks across the approaches set back from the intersection. Daniels et al. provide diagrams of different types of cycle facilities on roundabouts in the Netherlands [57]. Generally, entering traffic yields to traffic already in the intersection.

Traffic circle/rotary traffic island *	Raised concrete circles placed in the centre of minor street intersections; there are no splitter islands and the design vehicle is a passenger car.

Bicycle crossing	Distinct road crossings for cyclists that are sometimes raised or colored, and may have cyclist-operated traffic signals.

Bicycle box/advanced cycle stop line *	A right-angle extension to a bike lane at the head of an intersection, which allows cyclists to wait at the head of the traffic queue on a red traffic signal and then proceed through the intersection ahead of motor vehicle traffic on green.

Traffic diverter *	Bike-permeable barriers that require motor vehicle traffic to turn instead of traveling straight ahead through an intersection, or that prevent motor vehicles from entering a street.

* These types of infrastructure were not investigated in any of the studies identified for this review.

^† ^Terminology used in the "European Cycling Lexicon" (published by the European Economic and Social Committee at the Vélocity 2009 conference in Brussels). It gives a list of key cycling terms with corresponding photographs for cyclists and policy makers, in all 23 official European languages. It is freely available to download at: http://www.eesc.europa.eu/sections/ten/european-cycling-lexicon

**Table 2:** Studies that investigated relationships between bicyclist safety and intersection-related transportation infrastructure

Reference	Location; Design	Infrastructure types examined	Study population	Outcome measures	Analysis method	Control method	Effects observed
ROUNDABOUTS							

Schoon and Van Minnen (1994) The safety of roundabouts in the Netherlands [53]	The Netherlands; Observational, before-after intervention	Roundabouts vs. other intersection types; and roundabout design features	181 intersections before and after implementation of roundabouts	National database of bicycle and moped injuries and crashes* (529 before, 111 after)	Change in crash and injury rates after intervention.	Corrected for the temporal trends in crash and injury rates across all intersections in the Netherlands: national data that showed a 2 to 13% decrease over the study period. A seven-month "transitional period" following roundabout construction was not included in before-after analysis.	8% reduction in bicyclists' crash rate and 30% reduction in injury rate were observed following installation of new roundabouts. Among the 3 styles of roundabouts, those with cycle tracks had the greatest reductions in injuries to cyclists and moped users (90%), compared to those with no bicycle infrastructure (41% reduction) and those with a cycle lane (25% reduction).

Brüde and Larsson (2000) What roundabout design provides the highest possible safety? [54]	Sweden; Observational, non-intervention	Roundabouts vs. other intersection types	72 roundabouts with ≥ 100 cyclists/day	Police reports of 67 crashes*, 58 of which resulted in injuries	Comparison of observed and expected crash counts. Regression analyses to examine factors affecting crash counts and rates.	Calculated expected crashes and injuries using published prediction models for conventional intersections based on motor vehicle and bicycle traffic volumes.	At two-lane roundabouts, the observed crashes and injuries were more than twice those expected, whereas at single lane roundabouts there was no difference between expected and observed. Two other factors were associated with lower than expected crashes: single lane roundabouts with a central island radius > 10 m, and bicycle travel on bikeways rather than the roadway of the roundabout intersection.

Hels and Orozova-Bekkevold (2007) The effect of roundabout design features on cyclist crash rate [55]	Denmark - Odense; Observational, non-intervention	Roundabout design features	88 roundabouts	Police reports and Emergency Department records of 152 injuries*	Poisson regression and logistic regression analyses between cyclist injuries (3/year and probability, respectively) and roundabout characteristics: geometry, age, traffic volume (vehicles and cyclists), and location (urban/rural).	Adjusted for temporal changes in traffic volume.	In multiple regression, higher vehicle and cyclist traffic volumes and "drive curve" (a proxy for vehicle speed) were associated with higher numbers of cyclist crashes/year.

Daniels et al. (2008) The effects of roundabouts on traffic safety for bicyclists: An observational study [56]	Belgium - Flanders; Observational, before-after intervention	Roundabouts vs. other intersection types	91 intersections before and after implementation of roundabouts (40 inside built-up areas with speed limit of 50 km/h, and 51 in areas with speed limits of 70 or 90 km/h)	Police reports of 1060 injuries (411 at roundabouts, 649 at comparison intersections)	Effectiveness index = odds ratio for the before-after change in injury rates of the roundabout intersections as compared to the change in injury rates at conventional comparison intersections.	Comparison group: unchanged conventional intersections near intervention sites to account for temporal trends in safety and regression-to-the-mean (e.g. intersections may have been selected for roundabout construction because of higher numbers of crashes).	Roundabouts have the effect of increasing risk of crashes resulting in injury at or near the intersection (odds ratio = 1.27). The effect is stronger for intersections inside built-up areas (odds ratio = 1.48).

Daniels et al. (2009) Injury crashes with bicyclists at roundabouts: influence of some location characteristics and the design of cycle facilities [57]	Belgium - Flanders; Observational, before-after intervention	Roundabouts vs. other intersection types	Same data as Daniels 2008, above, except only 50 intersections in areas with speed limits of 70 or 90 km/h)	Same data as Daniels 2008, above.	Effectiveness index as Daniels 2008, above. Regression models to evaluate the roundabout design determinants of the effectiveness index.	Same data as Daniels 2008, above.	Roundabouts with cycle lanes had significantly higher risk (odds ratio = 1.93), whereas no increased risks were observed for roundabouts with mixed traffic, separate cycle tracks, or grade-separated paths. Roundabouts with 2 lanes and those replacing signalized intersections also had elevated risks.

BICYCLE CROSSINGS							

Gårder et al. (1998) Measuring the safety effect of raised bicycle crossings using a new research methodology [58]	Sweden - Gothenburg; Observational, before-after intervention	Bicycle crossings (raised above road level by 4-12 cm) vs. other intersection types	44 intersections (and 18.7 km of adjacent road sections) before and after implementation of raised bicycle crossings	Police or hospital reports of 287 crashes* (160 before, 127 after)	Calculated unadjusted number of crashes per month after intervention compared to before intervention.	Adjusted for traffic volume data collected on 2 intervention streets and 2 unchanged streets.	There was an 8% increase in crash frequency in the study area, but bicycle volume on these intervention sections grew by 50% more than unchanged streets - authors conclude that the intervention may have resulted in a safety improvement.

Jensen (2008) Safety effects of blue cycle crossings: a before-after study [59]	Denmark - Copenhagen; Observational, before-after intervention	Bicycle crossings (colored blue) vs. other intersection types	65 intersections before and after implementation of blue bicycle crossings	Police reports of 567 injuries (319 before, 248 after); 1,595 collisions (778 before, 817 after)	Comparison of observed injuries and crashes with expected (using fixed and random effects models).	Adjusted for temporal trends in traffic volumes and crashes, based on data from changed and unchanged intersections. Considered regression-to-the-mean, but no adjustment was deemed necessary.	Risk of crash/injury depends on number of colored crossings: 1 crossing = 10% reduction for injuries/19% for crashes; 2 crossings = 23%/48% increase; 4 crossings = 60%/139% increase. Authors hypothesize that non-intuitive findings may result from motorist confusion at sites with many crossings.

INTERSECTION DESIGN							

Wang and Nihan (2004) Estimating the risk of collisions between bicycles and motor vehicles at signalized intersections [60]	Japan - Tokyo†; Observational, non-intervention	Intersection design, including number of turn lanes, width of medians, pedestrian overpass	115 randomly selected signalized intersections with 4 legs	Police-reports of 585 bicycle-motor vehicle collisions	Three Poisson models of crash event risk: for "through" motor vehicle travel; left-turn travel; and right-turn travel.	Adjusted for average bicycle and motor vehicle volume, intersection location, speed limit, visual noise.	A higher number of turning lanes and presence of a wide median significantly increased risk of crash during motor vehicle turning maneuvers. Narrower entering approaches and wider medians increased crash risk in certain turning collisions. Increased cycle volumes were associated with lower collision risk with turning vehicles.

*These studies used only the term "accident" to describe crashes (collisions and/or falls) that may or may not have resulted in injury. We have substituted the words "crash", "collision" and/or "fall" based on our reading of the studies, as explained in the "Safety terminology" section of the text.

† In Japan, traffic drives on the left (so turns should be interpreted accordingly), and bicycles travel on sidewalks with pedestrians, not on the road.

**Table 3:** Studies that investigated relationships between bicyclist safety and transportation infrastructure related to roads, lanes and/or paths.

Reference	Location; Design	Infrastructure types examined	Study population	Outcome measures	Analysis method	Control method	Effects observed
ROADS, LANES AND PATHS							

Kaplan (1975) Characteristics of the Regular Adult Bicycle User [61]	United States; Observational, non-intervention	Major roads, minor roads, on-road bike routes or lanes, off-road (including bike paths and sidewalks)	3,270 cyclists who completed a survey distributed to a random sample of League of American Wheelmen members, geographically weighted to represent the population of each state.	Self-reporting (survey): 854 collisions or serious falls	Calculated crash rate per million miles for different infrastructure types, based on number of miles cycled and proportion of cycling on each type.	Adjusted for distance traveled.	Crash rates per million miles on major streets = 114, minor roads = 105, on-road bike routes or lanes = 58, and off-road = 292. Serious crash (involving emergency department visit or hospitalization) rates per million miles on major streets = 35, minor roads = 27, on-road bike routes or lanes = 25, and off-road = 77.

Lott and Lott (1976) Effect of Bike Lanes on Ten Classes of Bicycle-Automobile crashes in Davis, California [62]	United States - Davis; Observational, non-intervention	Roads with and without marked bike lanes	145 car-bike collisions	Police reports of 145 car-bike collisions	Comparison of numbers of collisions on roads with and without bike lanes, adjusting for neutral collision types.	"Neutral" collision types (considered to be independent of bike lane presence) used as method to adjust for car-bike traffic on the different road types. Neutral collision types defined as those where the cyclist or motorist failed to stop or yield, or the motorist made an improper left turn.	Bike lanes estimated to reduce collision frequency by 53%.

Smith and Walsh (1988) Safety impacts of bicycle lanes [63]	United States - Madison; Observational, before-after intervention	Major roads with and without marked bike lanes (one on left side of street, one on right side)	1.3-mile sections of 2 one-way arterial roads	City-maintained database of traffic crashes*: 87 crashes at study sites (1,411 crashes city-wide)	Compared crash counts per year before and after intervention.	Adjusted for average bicycle volumes city-wide in the before and after periods.	Increase in crash rates with bike lanes, especially for lane on left side of street in the initial year post-intervention. No statistically significant effect on long-term crash rates.

Tinsworth et al. (1994) Bicycle-related injuries: Injury, Hazard, and Risk Patterns [64]	United States; Observational, non-intervention	Major thoroughfares, neighborhood streets, sidewalks, bike paths, unpaved surfaces	(1) 420 cyclists who were injured and attended one of 90 emergency departments that report to the US Consumer Product Safety Commission, and (2) ~1250 other cyclists from a national probability sample	Hospital reports of 420 injuries (emergency department visits)	Multiple logistic regression, comparing infrastructure of injured cyclists (at location of injury event) and of cyclists from the national probability sample (infrastructure where cyclist rode more than 50% of the time).	Adjusted for hours of bicycle use per month, age, sex, size of community, daylight vs. dawn/dusk/night.	Relative risks (odds ratios) for injury by infrastructure type for adults: Major thoroughfares = 2.45; neighborhood streets (reference category) = 1; sidewalks = 1; bike paths = 0.14; unpaved surfaces = 0.11. Relative risks for children: neighborhood streets (reference category) = 1; sidewalks = 0.6; unpaved surfaces = 0.29; bike paths = 0.12.

Rodgers (1997) Factors Associated with the Crash Risk of Adult Cyclists [65]	United States; Observational, non-intervention	Roads, bike paths or lanes, off-road trails, other surfaces	2,978 cyclists who completed a survey (conducted by National Family Opinion for Bicycling magazine), including adults who purchased new bicycles, screened to match US population based on geographic region, population density, household income, household size, and age.	Self-reporting (survey): 280 respondents who had a crash or fell in the last 12 months	Multiple logistic regression comparing odds ratios for having a collision or fall versus not, according to primary riding surface of the cyclist.	Adjusted for miles traveled in warm weather months, age, sex, bicycle type, and geographic region of residence.	Odds ratios for risk of being a cyclist who had collision or fall in the last year, by primary riding surface, compared to roadway (= 1.0): bike path or lane = 0.60; other surfaces = 1.28; off-road trail = 7.17.

Moritz (1998) Adult Bicyclists in the United States: Characteristics and Riding Experience in 1996 [66]	United States; Observational, non-intervention	Major roads, minor roads, signed bike routes, on-street bike lanes, multiuse trails, off-road/unpaved trails, sidewalks	1,956 cyclists who completed a survey distributed to a random sample of League of American Bicyclists members, geographically weighted to represent the population of each state.	Self-reporting (survey): ~680 crashes	Relative danger indices calculated by dividing the proportion of crashes on a given infrastructure type by the proportion of commuting distance reported on that infrastructure. When index = 1.0, proportions of crashes and commuting distances are the same for that route type.	Adjusted for distance traveled.	Relative danger index by infrastructure type: major street without bike facilities = 0.66; minor street without bike facilities = 0.94; on-road bike routes = 0.51; on-road bike lanes = 0.41; multiuse trails = 1.39; off-road/unpaved trails = 4.49; "other" (mostly sidewalk) = 16.3.

Moritz (1998) Survey of North American Bicycle Commuters: Design and Aggregate Results [67]	United States; Observational, non-intervention	Major roads, minor roads, on-road bike routes & lanes, off-road bike paths, sidewalks	2,374 cyclists who completed a survey distributed via email lists, magazine advertisements, and word of mouth.	Self-reporting (survey): 271 serious crashes	Relative danger indices calculated by dividing the proportion of crashes on a given infrastructure type by the proportion of commuting distance reported on that infrastructure. When index = 1.0, proportions of crashes and commuting distances are the same for that route type.	Adjusted for distance traveled.	Relative danger index by infrastructure type: major street without bike facilities = 1.26; minor street without bike facilities = 1.04; on-road bike routes and lanes = 0.50; off-road bike paths = 0.67; "other" (mostly sidewalk) = 5.3.

Aultman-Hall and Hall (1998) Ottawa-Carleton commuter cyclist on- and off-road incident rates [68]	Canada - Ottawa; Observational, non-intervention	Roads, off-road paths, sidewalks	1452 commuter cyclists who completed a survey distributed on parked bicycles.	Self-reporting (survey): 187 injuries, 194 collisions, 234 falls	Event rates calculated per distance traveled on each infrastructure type based on GIS analyses of mapped commuting routes; relative risks for the three infrastructure types compared using Poisson distribution and Hauer statistical test.	Adjusted for distance traveled. Also adjusted (via weighting) for differences in use of various infrastructure types by cyclist characteristics: weekly commute distance; left turning method; comfort on busy streets; and belonging to a cycle club or having taken a training course.	Compared to cycling on-road, there were no differences in collision rates for off-road or sidewalk cycling, but the relative risks of falls were 2.1 for off-road paths and 4.0 for sidewalks, and of injury were 1.6 for off-road paths and 4.0 for sidewalks.

Aultman-Hall and Kaltenecker (1999) Toronto bicycle commuter safety rates [29]	Canada - Toronto; Observational, non-intervention	Roads, off-road paths, sidewalks	1196 commuter cyclists who completed a survey distributed on parked bicycles.	Self-reporting (survey): 182 injuries, 300 collisions 203 falls	Event rates calculated per distance traveled on each infrastructure type based on GIS analyses of mapped commute routes; relative risks for the three infrastructure types compared using Poisson distribution and Hauer statistical test.	Adjusted for distance traveled. Also adjusted (via weighting) for differences in use of various infrastructure types by cyclist characteristics: age; sex; weekly commute distance; and comfort on busy streets.	Compared to cycling on-road, relative risks of collisions were 3.5 for off-road and 2.0 sidewalk cycling, of falls were 1.5 for off-road paths and 9.0 for sidewalks, and of injury were 1.8 for off-road paths and 6.4 for sidewalks.

ROAD DESIGN CHARACTERISTICS							

Klop and Khattak (1999) Factors Influencing Bicycle Crash Severity on Two-Lane, Undivided Roadways in North Carolina [69]	United States - North Carolina; Observational, non-intervention	Characteristics of 2-lane undivided roads: curve vs. straight; level vs. grade; right shoulder width; intersection or not; street lighting	1,025 collisions with motor vehicles.	Police reports of bicycle collisions (recorded in the Highway Safety Information System) identifying injury severity†. Classified as property damage only, pain, non-incapacitating, incapacitating, and fatal.	Multivariate ordered probit model comparing the 5 levels of injury severity.	Adjusted for traffic volume, speed limit, year, rural-urban, weather, daylight.	More severe injuries were significantly associated with the following infrastructure characteristics: grades on both curved and straight roads; and unlit roads at night. Other factors associated with higher injury severity included: higher speed limits; lower average annual daily traffic; and fog.

Allen-Munley et al. (2004) Logistic model for rating urban bicycle route safety [70]	United States - Jersey City; Observational, non-intervention	Width and grade of roads, one-way versus two-way road configuration, highway versus non-highway road type	314 injuries resulting from collisions with motor vehicles.	Police reports of 314 bicycle crashes, identifying injury severity†. Classified as property damage only, minor and serious.	Ordinal logistic regression comparing the three levels of injury severity.	Adjusted for whether child or adult, traffic volume per lane, household income, population density, land use, weather, and daylight.	More severe injuries were significantly associated with wider roads, perceptible grades, and one-way streets, pavement not resurfaced in last 10 years, and highway road type (the first three variables at p < 0.05, the latter three at p < 0.10).

ROAD SURFACES							

Rivara et al. (1997) Epidemiology of bicycle injuries and risk factors for serious injury [16]	United States - Seattle; Observational, non-intervention	Surface type: paved vs. unpaved	3390 injured cyclists who completed a questionnaire about demographic characteristics, cycling experience, crash circumstances, and helmet use and fit.	Emergency department, hospital and medical examiner records of injuries, classified using the injury severity† score (ISS)	Univariate and multivariate logistic regression comparing cyclists with severe injuries (ISS > 8) to those with less severe injuries.	Adjusted for age, motor vehicle involvement, speed, helmet use.	Decreased risk of severe injury on unpaved surfaces (odds ratio = 0.7, not statistically significant). Motor vehicle involvement was strongest risk factor (odds ratio = 4.6).

SIDEWALKS							

Wachtel and Lewiston (1994) Risk-factors for bicycle motor-vehicle collisions at intersections [71]	United States - Palo Alto; Observational, non-intervention	Sidewalks vs. roadways	89 bicycle-motor vehicle collisions at intersections or junctions on three major arterial roads.	Police reports of 89 collisions	Relative risk of collisions for cyclists on sidewalks vs. on roadway. Risk calculations used 8-hour bicyclist counts at 9 intersections (7 signalized and 2 with stop signs) on the 3 arterials.	Adjusted for age (whether child < 18 or adult), sex, and direction of travel (with or against motor vehicle traffic).	Cycling on the sidewalk is associated with higher risk (RR = 1.8). The elevated risk on sidewalks is almost exclusively related to cycling against traffic (RR = 1.9) vs. with traffic (RR = 0.9).

STREET LIGHTING							

Kim et al. (2007) Bicyclist injury severities in bicycle-motor vehicle crashes [72]	United States - North Carolina; Observational, non-intervention	Street lighting, straight versus curved roadway, street configuration (one-way, two-way, divided or not)	2934 injuries resulting from collisions between a single motorist and a bicyclist.	Police reports of injury severity†. Classified as fatal; incapacitating; non-incapacitating; possible or no injury.	Multinomial logit model, comparing the probability of four injury severity outcomes	Adjustment for all factors included in model: bicyclist age, intoxication, helmet use; driver intoxication; vehicle speed and type; crash characteristics including fault and directions of travel; land use; time of day; weather.	Infrastructure-related determinants that increased the probability of severe injury in an crash were: unlit roads at night; curved road geometry; and undivided street configuration.

Wanvik (2009) Effects of road lighting: an analysis based on Dutch crash statistics 1987-2006 [73]	The Netherlands; Observational, non-intervention	Road lighting on rural roads	~125,000 bicycle crashes resulting in injury from 1987-2006.	Police reports of ~125,000 injuries	Odds ratio estimating risk of crash in darkness versus daylight on lit versus unlit roads.	Adjusted for hour of the day, darkness, and season, by summing log odds ratios calculated separately for these factors. Log odds ratios were weighted in inverse proportion to the variance of the odd ratio.	Presence of lighting on rural roads reduces bicyclist injuries by ~60%.

* This study used only the term "accident" to describe crashes (collisions and/or falls) that may or may not have resulted in injury. We have substituted the words "injury", "crash", "collision" and/or "fall" based on our reading of the studies, as explained in the "Safety terminology" section of the text.

† Injury severity does not reflect risk of an incident, but rather the outcome of the incident once it occurs.

## Ridership and Safety

North Americans remain less likely than Europeans to choose bicycle transport for either short or long trips. In part, this may be due to differences in urban form between North American and European cities, particularly density and interconnectedness [25], but perceived and actual injury risks are also important.

Data on the share of trips made by bicycle are not often directly comparable between jurisdictions owing to differences in the survey methods employed (e.g. sampling scheme, definition of a trip, etc.), but comparisons are typically justified by the inability of these methodological disparities to explain the substantial difference observed (e.g. about 1% of trips are made by cycling in North America vs. an estimated 10% of trips in Switzerland, Germany, Austria, Sweden, Finland, and Belgium (Flanders), and more than 20% of trips in Denmark and the Netherlands) [26]. Along with these lower cycling rates, there is also a higher risk of injury associated with cycling in North America: an analysis of traffic injuries indicated a two to three fold higher risk of death and an eight to 30 fold higher risk of injury while cycling in the United States vs. Holland and Germany, using either of the traditional transportation denominators: per trip or per kilometer traveled [27]. While these comparisons underscore cycling injury risks, they also provide reason for optimism. If cycling is safer in European cities, it can be made safer in North America.

There are clearly bicycling safety and popularity "gaps" between (and within) Europe and North America [28]. In addition, there is an important safety gap between cyclists and other transport modes: estimates from both continents suggest that cyclists are seven to 70 times more likely to be injured, per trip or per kilometer traveled, than car occupants [27,29]. It is likely that public perception of a lack of safety acts as a deterrent to cyclists in North America: in surveys asking about factors that affect the choice of cycling as a mode of transportation, concern about safety is one of the most frequently cited deterrents [[30-34], and Winters M, Davidson G, Kao D, Teschke K: Motivators and deterrents of bicycling: factors influencing decisions to ride, submitted]. For example, in a survey of adults in the Vancouver metropolitan area, the following were among the top deterrents: the risk of injury from car-bike collisions; the risk from motorists who don't know how to drive safely near bicycles; motorized vehicles driving faster than 50 km/hr; and streets with a lot of car, bus, and truck traffic [33]. The good news is that there is evidence that perceived safety improvements in bicycle transportation have an aggregate elasticity value greater than one (i.e. a 10% increase in perceived safety results in greater than 10% increase in the share of people commuting by bicycle) [32].

Increased ridership rates may result in improved safety for cyclists: injury rates have been shown to decrease with increased cycling rates. This principle of "safety in numbers" is supported by studies of injury and ridership patterns in California, Australia, and Europe, as well as between cities and within cities over time [35-38]. There are a number of potential explanations. Motor vehicle drivers may not expect cyclists when there are few of them on the roads, and thus make so-called "looked-but-failed-to-see" errors that can result in collisions [39]. When motorists and cyclists are unaccustomed to sharing the road, both parties may hold incorrect assumptions about what the other party will do [40]. Increased cycling rates may mean that more motorists also use bicycles as a mode of transport, making motorists more attuned to cyclists and their movements, and encouraging them to drive in a way that accounts for potential interactions [36]. Finally, a larger cycling population means stronger lobbying power for cycling resources.

Finally, it is worth considering long-term temporal trends in motor vehicle injuries. The injury rate from motor-vehicle crashes has steadily declined since the 1920s in many parts of the world, in part attributable to improvements in road-related infrastructure [41]. This provides reason for optimism: the risk of injury or death from traffic crashes is modifiable, and this is likely to extend to the infrastructural determinants of cycling injuries.

## Safety and Infrastructure Terminology

### Safety terminologyy

Bicycling safety is usually quantified by measuring one or more of the following metrics: injuries; crashes; and conflicts. Injuries may include fatalities and can be classified according to their type and severity using standardized methods such as the World Health Organization's International Classification of Diseases (ICD) [42] and the Association for the Advancement of Automotive Medicine's Abbreviated Injury Scale (AIS). Crashes can be classified as either a collision or a fall, where a collision is defined as an event in which the bicycle hits or is hit by any other object, regardless of fault, and a fall is an event (not caused by a collision) where the bicycle and/or bicyclist lands on the ground.

A conflict is normally defined as an interaction between a bicyclist and another road user such that at least one of the parties has to change speed or direction to avoid a collision. Types of conflict examined in bicycling safety studies include avoidance maneuvers at intersections [43-45], bicycle-motor vehicle interactions during passing events on roads, lanes, or paths [46-49], and "wrong side passing events" on multi-use paths [50]. Conflict studies may offer valuable insights into how cyclists and other road users behave during their interactions on various types of transportation infrastructure. However, it is not possible to determine whether the safety of the cyclists was compromised during the conflict events. In addition, the conflict studies we identified were generally based on a small number of observed events, which were made over a limited time period (usually several hours), and often in a single geographical location. Therefore, papers that used conflict as their sole outcome measure have not been included in this review.

In the literature that examines traffic-related injuries and crashes (including many of the papers reviewed here) the word "accident" is frequently used, for example in the phrase "motor vehicle accident". However it has been argued that the term "accident" implies that the event in question has happened entirely by chance, and is therefore unpredictable and unpreventable [51] as opposed to being a result of modifiable risk factors. The editors of BMJ have even gone as far as to ban the use of the term [52]. We have refrained from using the word "accident" in this review, instead using the more specific terms "incident", "injury", "crash", "collision" and "fall" as appropriate. However, we do indicate if the original study authors used the word accident to describe the outcome measure.

### Infrastructure terminology

Key terms that describe transportation infrastructure used by cyclists are defined in Table 1. We have indicated if a given type of infrastructure was not studied in the English-language scientific literature identified by our search.

## Methods

### Search strategy

We searched the following bibliographic databases: Pubmed and Medline, which index over 3,600 international medical and health care journals (1949 to present); Web of Science, which includes the Science Citation Index, the Arts and Humanities Citation Index, and the Social Sciences Citation Index (1989 to present); and Transportation Research Information Services, which includes references to books, technical reports, conference proceedings and journal articles in the field of transportation (1960 to present). In order to identify relevant studies, we used search terms related to the safety of bicyclists, and to transportation infrastructure. Combinations of the following keywords were used in the searches, (with "wildcards" used where appropriate to capture variants on terms, e.g. bicycl*): bicycle, safety, injury, accident, crash, conflict, infrastructure, road, and intersection. Reference lists of all relevant papers including review papers were searched as a source of additional citations. The initial literature search was conducted in summer 2008 and updated through to June 2009.

### Inclusion and exclusion criteria

All papers identified by the search were initially screened for relevance using the title and/or abstract. Specifically, we sought papers that met the description of injury epidemiology studies, injury severity studies, and crash/collision/fall rate studies, and that considered some aspect of infrastructure as a determinant/predictor of bicyclists' safety. These included "before and after" studies that examine the safety impact (change in injury or crash rate for cyclists) of some infrastructural change. Those papers considered potentially relevant were collected, and the full text versions were then further reviewed for relevance.

Papers were considered relevant and included in the review if they met the following criteria:

• they investigated a relationship between transportation infrastructure (designed for either motorized or non-motorized use) and a clearly-defined metric of bicyclist safety (injury, injury severity, crash/collision/fall); and

• they were English-language publications describing empirical research conducted in an Organisation for Economic Co-operation and Development (OECD) country. For countries outside the OECD, it was expected that the transportation infrastructure and bicycling rates (as well as the socio-economic motivators of bicycling) would be different, and consequently the study results may not be applicable across regions. The literature search did not locate any relevant papers describing studies conducted outside the OECD.

We excluded papers from further review if they met any of the following criteria:

• studies of injuries or crashes that occurred when the bicycle was being used for bicycle racing, "off-road mountain-biking", trick/trials riding, or play;

• studies only examining non-infrastructural determinants of safety such as helmet-use, bicycle type, personal characteristics of the bicyclists or motor vehicle drivers (e.g. age, sex, experience);

• studies of injuries not related to a crash event, e.g. chronic injuries related to riding position;

• studies examining gross numbers/types of injuries in a region for a given time period, without either calculating rates (per exposure/riding time) or considering infrastructural determinants of those injuries;

• studies that reported only subjective perceptions of safety or risk, whether by lay-public or experts; and

• studies that examined only "conflict" between cyclists and other road users (refer to the section on "safety terminology"), but where crashes or injuries were not identified.

## Results

In total, 23 papers were identified that met the inclusion criteria. Eight examined infrastructure related to intersections, and are abstracted in detail in Table 2[53-60]. Fifteen papers examined infrastructure related to "straightaways", i.e. roads, lanes, paths, etc., and are abstracted in Table 3[16,29,61-73]. Studies are presented in the tables first by type of infrastructure, then by year for each type.

Ten of the 23 studies reviewed used injuries (or both injuries and crashes) as a metric of bicyclist safety, four examined injury severity, and nine examined crashes (i.e. collisions and/or falls). Most of the studies were published since 1994, except two US studies which were published in the mid-70s [61,62] and one which was published in 1988 [63]. All the study designs were observational. Five of the intersection-related papers [53,56-59], but only one of the road/lane/path-related papers [63], were "before-after" studies that quantified the change in cyclist safety before and after some infrastructure-related intervention took place. The remaining papers were classified as "non-intervention" observational studies. Most of the studies based their analyses and conclusions on at least 150 observations of injury or crash events, and seven studies based their analyses on more than one thousand observations. However one study of roundabouts examined only 67 crashes, 58 of which resulted in injuries [54], and two non-intersection studies examined 87 and 89 crashes on roads with and without marked bike lanes [63], and on sidewalks versus roads [71] respectively.

Thirteen of the studies were published in public health related journals (mainly Accident Analysis & Prevention and the Journal of Safety Research), and nine were published in transportation engineering journals (mostly Transportation Research Record). The remaining study (on the safety of different road/lane/path infrastructure types) was conducted as part of a Master of Science thesis [61].

All but one of the studies about intersection-related infrastructure (Table 2) were conducted in European countries. Five of the European intersection-related studies examined the safety of roundabouts and two examined marked bicycle crossings. The non-European study examined how intersection design in Japan influenced number of bicycle-motor vehicle collisions [60]. Cyclists in Japan are required by law to travel on the sidewalk, so the results from this study may not be generalizable to countries with different traffic rules.

The findings of the roundabout studies show some consistency, with elevated risks for cyclists after installation of roundabouts with multiple traffic lanes or with marked bike lanes, whereas there were risk reductions or no apparent increase in risk at roundabouts with separated cycle tracks [54,56,57]. One study showed a decreased risk for cyclists and moped riders after installation of roundabouts in the Netherlands [53], but the authors did not disaggregate the results for these two road-user groups. The finding from this study - that roundabouts with separated cycle tracks had a greater safety effect than those with on-road marked bike lanes or no bicycle infrastructure - is consistent with other research. Another study on roundabout safety in Flanders found a similar effect for "vulnerable road users" [74], but we have not included this study in our table because the vulnerable road user population included pedestrians and motorized two-wheeler riders as well as cyclists. It is likely that the safety effect of roundabouts, as measured in such "before-after" studies, will depend on the "before" configuration of the intersections in question.

The two studies of the safety effect of marked bicycle crossings at intersections looked at different design aspects (one on physically elevated crossings, one on colored crossings) and did not provide clear conclusions. Although the study on elevated crossings showed a small increase in the number of crashes after the crossing was installed, the bicycle traffic volume grew by 50% on the streets after the intervention, as compared to unchanged streets in the area, and this was not adjusted for in the analysis [58]. The second study showed a reduction in injury or crash risk when there was one colored bicycle crossing at an intersection, but an increase in injury or crash risk when there were two or more colored crossings [59].

Of studies examining infrastructure related to straightaways on roads, lanes, and paths (Table 3), all but one were conducted in Canada or the US. The only European study in this category is very different in its focus: the safety effect of rural street lighting in the Netherlands [73]. Perhaps unsurprisingly, that study found that the presence of street lighting on rural roads reduced the rate of cyclists' injuries by half. The effect was corroborated by an injury severity study that found that crashes resulting in more severe injuries were significantly associated with unlit roads at night [69].

Most of the remaining studies in this category compared cyclist injury or crash rates on different types of road- or path-related infrastructure that cyclists commonly travel, namely major and minor roads without specific cycling facilities, roads with wide curb lanes or marked bike lanes, on-road bike routes, off-road bike-specific or multi-use paths, and sidewalks. A difficulty with this literature was that several facilities (between two and seven in number) were grouped into categories, such that facilities with potentially different risks were classified within a single category. In addition, the categorizations differed from study to study, and the terminology used was sometimes not clearly defined or consistently used. Despite these limitations, there are still some consistent messages.

On-road marked bike lanes were found to have a positive safety effect in five studies, consistently reducing injury rate, collision frequency or crash rates by about 50% compared to unmodified roadways [61,62,65-67]. Three of those studies [61,66,67] found a similar effect for bike routes. One study [63] found that there was an increase in crash rates in the year following installation of marked bike lanes on a major road, especially for a section counter to on-road traffic flow, but this effect was not sustained over the long term.

There is less consistent evidence about off-road riding, possibly because this category encompassed a wide variety of facility types. There may have been confounding factors such as whether the surface was paved or unpaved, or for bicycles only or multiple user groups. Two studies examined off-road bike paths and found reduced risks, ranging from 0.11 to 0.67 times the risk of cycling on minor roads [64,67]. Two studies that grouped paved and unpaved, bicycle only and multi-use urban trails in their off-road path category found elevated risks, 1.6 to 3.5 times higher than riding on-road [29,66,68]. Studies that examined unpaved off-road trails as a separate category found risks of injury 2.5 to 7.2 times higher than on-road cycling [61,65,66] and 8 to 12 times higher than bike routes, lanes, or paths [65,66].

Most studies that considered sidewalk-riding suggested that it is particularly hazardous for cyclists, with estimates of 1.8 to 16 times the risk of cycling on-road [29,66-68,71]. However one study found that the risk of traveling on the sidewalk was the same or lower than riding on residential streets [64]. Another considered the direction of travel and found that the elevated risk when sidewalk cyclists entered intersections was almost exclusively related to cycling against the flow of adjacent on-road traffic [71].

Four studies examined the association between various infrastructural characteristics and injury severity [16,69,70,72]. More severe injuries were significantly associated with motor vehicle involvement, unlit roads at night, wider roads, perceptible road grades, and one-way streets. Injury severity does not reflect risk of an incident, but rather the outcome of the incident once it occurs. In comparison, the studies that examined injury or crash rates, as opposed to those that concentrated on injury severity, were our primary focus since we are most interested in shaping transportation infrastructure for injury prevention.

## Discussion

In this review we have described two categories of infrastructure: the first related to intersections; and the second related to straightaways on roads, lanes, and paths. It is of interest to note that studies of the former type of infrastructure were conducted almost entirely in Europe, while studies of the latter were conducted almost entirely in North America. The reason for this may be the substantial differences in urban form, existing cycling infrastructure, cycling rates, and even the culture of cycling between Europe and North America. Pucher and colleagues have discussed this issue extensively [26,75]. There is also significant variety in infrastructure design from one country to another, and even within a given city. Despite this, our review has revealed that relatively few types of infrastructure have been studied. For example, some common types of infrastructure in North American cities have not been assessed: traffic circles; bike boxes; sharrows; speed bumps/humps; and traffic diverters (Table 1). In addition, except for studies of roundabouts, we did not find any injury or crash studies that investigated cycle "tracks", a bicycle-specific design that is frequently available in high modal share European cities. One of the limitations of this review is that we have only included studies in the English scientific literature, although we are aware that there may be studies reported only in other (particularly European) languages.

The principal trend that emerges from the papers reviewed here is that clearly-marked, bike-specific facilities (i.e. cycle tracks at roundabouts, bike routes, bike lanes, and bike paths) were consistently shown to provide improved safety for cyclists compared to on-road cycling with traffic or off-road with pedestrians and other users. Marked bike lanes and bike routes were found to reduce injury or crash rates by about half compared to unmodified roadways. The finding that bicycle-specific design is important applies also to intersections with roundabouts, where it was found that cycle tracks routing cyclists around an intersection separately from motor vehicles were much safer than bike lanes or cycling with traffic. It has been suggested that the reason for high rates of bicycle-motor vehicle collisions at intersections is that motor vehicle drivers may be making "looked-but-failed-to-see" errors, whereby they search for oncoming motor vehicles but do not recognize that a cyclist is approaching because they are not looking for them [39,40].

Although roundabouts at intersections are not common in North America, they are relatively popular in many European countries. It is possible that they may see more widespread use in North America in the future because of evidence that conversion of intersections to roundabouts reduces crash risk for motor vehicle road users by 30-50% [76], especially when they replace intersections that were not previously signal-controlled. However, because the cyclist-specific safety effect of roundabouts appears to be highly dependent on their design, transportation infrastructure planners should carefully consider interactions between cyclists and other traffic modes. A literature review on the safety effect of roundabouts, prepared for the 18th Workshop of the International Co-operation on Theories and Concepts in Traffic Safety [77], came to similar conclusions. It may be prudent to avoid installing roundabouts in areas where there is a high proportional volume of bicycle traffic, for example along designated bicycle routes on residential roads. In some North American cities there is retrofitting of "traffic circles" at intersections in residential areas. Since these are quite different from the larger-diameter roundabouts found in Europe, their effect on cyclist safety should be investigated before more widespread use is advocated.

The reviewed literature also confirms some things that may already be "common-sense" for transportation planners and safety experts: that streets used by cyclists at night should have good street-lighting, road surfaces should be paved and well-maintained, and bike routes should avoid excessive grades wherever possible.

An issue with the literature to date, especially that related to roads, lanes, and paths, is that some investigators did not define the terminology used. For example, the meaning of bike "path" was not defined in the paper by Tinsworth et al. [64]. Other investigators clearly defined their infrastructure terms, but grouped facilities that may have different injury risks. For example, the studies of Aultman-Hall et al. [29,68] defined paths as "an off-road (usually multi-use) paved or unpaved path or trail," grouping paths for bikes only, which were found by others to have lower risks than cycling on roads [64,67], with unpaved trails, which were found by others to have higher risks [61,65,66]. Definitions of terminology are especially important in questionnaire-based studies to ensure that study participants are all answering with the same infrastructure in mind; photos can be helpful in this regard [33].

Clear and specific categorization is also vital to transportation planners and engineers, so they can distinguish sometimes subtle differences between successful and problematic design characteristics. One of the difficulties of the studies in the English-language literature to date is that the range of infrastructure studied is small compared to the range of configurations used between and within jurisdictions. Some examples are described above, but there are many other features that merit investigation: stop signs; numbers of roads intersecting; junctions such as driveways and lanes; cyclist lane of travel in relation to parked cars; surface features such as cobble stones or street-car (tram) tracks; traffic calming measures such as diverters or road humps; and road/lane/path curvature.

Underreporting of some events is an issue that is common to all studies of bicycle injuries and crashes. Many of the studies reviewed here relied on administrative data sources including hospital records [16,62,64], police reported accidents [54-61,69-73], and national or city-maintained registries [53,63], all of which are likely to miss less severe events. For example, one of the large surveys [67] found that 9.8% of the respondents had had a crash in the last year, but only two in five crashes (38.2%) had been reported to police. Over half (56.6%) required medical attention, but only one in twenty crashes (5.5%) required admission to a hospital. This underreporting may create bias in infrastructure-specific risk calculations, since collisions involving motor vehicles may be more likely to be reported to police for insurance reasons and to hospitals because they are more severe, as compared to collisions that happen with non-motorized users (which may happen more frequently on off-street paths). Results of studies using these data sources should be interpreted as reflecting risk of severe events. Other studies in this review used data from cyclist surveys that may capture a wider range of crash types, including those that are less severe [29,61,65-68]. However, survey data will not capture events that resulted in fatalities (though these are extremely rare) or catastrophic incapacitating brain, spinal cord or other injuries and, depending on the method of survey administration, may not capture individuals who no longer cycle following a crash [29,68]. No single study design can overcome these reporting problems, thus the importance of looking for consistency of results across different designs.

A great challenge in studying cycling injuries is ensuring that comparisons control for the number of cyclists at risk (also called "exposure to risk"). The before-after studies reviewed here aimed to do this by comparing numbers of injuries on the same intersection or roadway prior to and post introduction of an infrastructure intervention, with the assumptions that underlying traffic levels, injury rates, and types of cyclists stay the same. These assumptions may not hold [58], so some of these studies also adjusted for temporal trends in traffic volumes [58,59,63] or injury rates in the area [53], or made additional comparisons to unchanged intersections [56-59]. The non-intervention studies needed to include methods to derive bicycling trip volumes on the infrastructure types being compared. Sometimes these came from administrative data collected by transportation authorities [54,55,60,71,73], and sometimes from study participants describing the route of an injury trip or their typical cycling location [29,61,64-68]. Injury severity studies made comparisons within the injured populations, so did not require trip volume denominators [16,69,70,72], but this meant that they examined differences in severity of the outcome once in an injury event, not the original risk of the event itself.

Though the most basic requirement for studies examining risk of crashes or injuries is to account for exposure to risk, there are many other factors that may confound comparisons and that ideally would be controlled in study design or adjusted for in analyses. For example, men and women or people in different age groups may choose to cycle on different facility types, and might have different skill levels or risk-taking behavior, thus creating the potential for confounding associations between infrastructure and injury. While it is difficult to control for all potential confounders, many of the non-intervention studies reviewed here did adjust for personal factors such as age [16,29,64,65,70,71], sex [29,64,65,71], cycling experience [29,68], bicycle type [65], and environmental factors such as time of day [64,69,70,72,73] and weather [65,69,70,72]. Most injury severity studies adjusted for helmet use [16,69,72]. A style of observational study that can control for most potential confounders is the case-crossover design [78]. Such a study is underway in the Canadian cities of Toronto and Vancouver. It will compare infrastructure at the injury site to that of randomly selected control sites on the same trip, thus within-trip factors (including age, sex, cycling experience, propensity for risk taking, alcohol or drug use, bicycle type and condition, visibility via clothing or bicycle lights, weather, time of day, etc.) are controlled in the design.

## Conclusion

Although the effect of infrastructure design on cyclist safety was first studied more than three decades ago, the literature on the topic remains remarkably sparse. This review highlights opportunities for more detailed and controlled studies of infrastructure and cycling injuries.

The evidence to date suggests that purpose-built bicycle-only facilities (e.g. bike routes, bike lanes, bike paths, cycle tracks at roundabouts) reduce the risk of crashes and injuries compared to cycling on-road with traffic or off-road with pedestrians. Street lighting, paved surfaces, and low-angled grades are additional factors that appear to improve cyclist safety. The major advantage of infrastructure modifications, compared to helmet use, is that they provide population-wide prevention of injury events without requiring action by the users or repeated reinforcement. Given the influence of safety on individuals' decisions to cycle, the importance of cycling modal share to safety, and the ancillary benefits of this active and sustainable mode of transportation, infrastructure enhancements have the opportunity to promote an array of improvements to public health.

## Abbreviations

OECD: Organisation for Economic Cooperation and Development; km: kilometer; ICD: International Classification of Diseases; AIS: Abbreviated Injury Scale.

## Competing interests

The authors are part of a research team that is studying the association between bicyclists' injuries and the cycling environment in Vancouver and Toronto. The Heart and Stroke Foundation of Canada and the Canadian Institutes of Health Research have funded this three-year study. See http://www.cher.ubc.ca/cyclingincities/injury.html for more information.

## Authors' contributions

KT, CR, AH and PC conceived of the study and developed the literature search strategy. CR and AH conducted the literature search. CR, AH, MW, and KT reviewed the included papers, and abstracted them for the detailed tables prepared for this paper. CR wrote the initial draft of the manuscript, and the other authors all contributed to its development, particularly the discussion. All authors reviewed and approved the final manuscript.
